# Role of the Antioxidant Lycopene in Bone Health: Emphasis on the RANKL/RANK/OPG Signaling Pathway

**DOI:** 10.1002/fsn3.72229

**Published:** 2026-08-13

**Authors:** Helia Esmaili, Nazanin Asgari, Negar Mosayeb, Sadegh Jafarnejad

**Affiliations:** ^1^ Research Center for Biochemistry and Nutrition in Metabolic Diseases Kashan University of Medical Sciences Kashan Iran

**Keywords:** bone, bone remodeling, lycopene, osteoporosis, RANKL/RANK/OPG signaling pathway

## Abstract

Bone health is maintained by a balance between bone formation and bone resorption. Lycopene, a lipophilic carotenoid and an acyclic isomer of beta‐carotene, functions as an antioxidant and may improve bone health via multiple pathways, including the suppression of inflammatory cytokines, stimulation of osteoblast activity, regulation of the wingless‐related integration site (Wnt)/β‐catenin pathway, and modulation of the receptor activator of nuclear factor‐kB (RANK)/RANK ligand (RANKL)/osteoprotegerin (OPG) pathway. Our understanding of the mechanisms involved in bone remodeling has improved with the discovery of the RANK/RANKL/OPG pathway. Elevated RANKL/RANK signaling promotes osteoclastogenesis and consequently bone resorption, while inhibition of this pathway may prevent bone resorption. This review summarizes current evidence on the impact of lycopene on bone health via the RANK/RANKL/OPG signaling pathway, a key regulator of bone remodeling.

## Graphical abstract

Bone health may be maintained through several interconnected mechanisms attributed to lycopene including its potent antioxidant, anti‐inflammatory, osteogenic, and anti‐resorptive actions. Lycopene may protect osteoblasts by reducing reactive oxygen species and stress‐mediated apoptosis. Furthermore, lycopene may attenuate inflammation by suppressing NF‐κB signaling and reduction of pro‐inflammatory cytokines, including TNF‐α, IL‐1β, IL‐6, and IL‐17. Increased osteoblastogenesis and enhanced osteoid formation may occur via increased expression of RUNX2, type I collagen, alkaline phosphatase, and osteocalcin with the implication of Wnt/−catenin as well as ERK1/2 signaling. Concurrently, suppression of osteoclast formation and bone resorption is demonstrated through alteration of RANKL/RANK/OPG balance, namely reduction of RANKL and elevation of OPG. In diabetes, lycopene may, in addition, facilitate bone turnover renewal, thereby preserving bone metabolic balance. Alterations of connexin 43‐mediated gap‐junction communication, induction of phase‐II detoxifying enzymes, interactions with other nutrients such as vitamin D, interactions with other carotenoids, and retinoid‐dependent signaling regulation may offer additional explanations of lycopene actions. Overall, these complementary mechanisms suggest that lycopene may help maintain bone homeostasis by limiting oxidative and inflammatory stress while promoting bone formation and restraining bone resorption through modulation of multiple pathways, including RANK/RANKL/OPG.

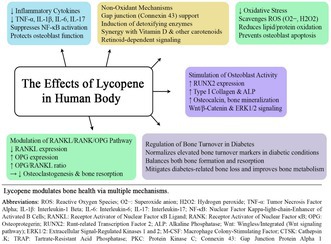

## 1 Introduction

Bones are vital living organs composed of approximately 70% minerals and 30% organic material (Vannucci et al. 2018). Human skeletal bones act as the sites of attachment for muscles, tendons, and ligaments, aiding locomotion (Salhotra et al. 2020). Osteoporosis results in adverse effects on bone mass and architecture, heightening the risk of fracture with age (Elias et al. 2025).

In the United States, the prevalence of osteoporosis is expected to increase 2.4‐fold in females and 3.1‐fold in males by 2050 (Seem et al. 2019). Bone consists of various cell types, such as osteoclasts, osteoblasts, osteocytes, and bone lining cells (Cai et al. 2025). Osteoblasts are mainly derived from mesenchymal stem cells (MSCs). Osteoclasts are further derived from hematopoietic stem cells (HSCs) of the monocyte/macrophage lineage (Thompson et al. 2012). The osteoclast is a multinucleated cell mediating bone resorption (Kylmäoja et al. 2018).

On the other hand, osteoblasts generate macrophage colony‐stimulating factor (M‐CSF), a significant factor for osteoclast development, thereby controlling osteoclastogenesis (Sun et al. 2021). While osteoblasts lay new matrix in bones, causing bone modeling and remodeling, osteoclasts mostly resorb mineralized bone matrix (Boyce and Xing 2008). Disruption of this equilibrium might result in conditions like osteoporosis and fragility fractures (Nardone et al. 2014).

RANKL and OPG belong to the tumor necrosis factor (TNF) superfamily and its related TNF receptor (TNFR) family. Their interaction with the RANK is crucial for regulating osteoclast production, activation, and survival, both in normal bone modeling and remodeling, as well as in several pathological situations linked to amplified bone turnover (Silva and Branco 2011). The RANK/RANKL/OPG system, as a sophisticated signaling network, rigorously regulates bone modeling and remodeling by controlling osteoclast formation, activation, and survival. RANK, a receptor on osteoclast precursors and mature osteoclasts, binds to the RANKL ligand produced by osteoblast‐lineage and stromal cells. Osteoclast differentiation and activation are driven by RANKL signaling, and the balance between RANKL and its decoy receptor, osteoprotegerin (OPG), ultimately determines the extent of bone resorption (Simonet et al. 1997; Yasuda et al. 1998). As a competitive decoy receptor for RANKL, OPG reduces osteoclast formation and activity by preventing RANKL from binding to RANK (Zhang et al. 2022). The binding of OPG to RANKL modulates activation, formation, and survival of osteoclasts during normal bone formation and resorption, and also regulates various pathological conditions identified by increased bone turnover (Silva and Branco 2011; Yasuda et al. 1998).

Investigating the molecular mechanisms behind these imbalances has driven the development of novel treatments for osteoporosis. Bisphosphonates, which inhibit bone resorption, are one example of medical interventions that have revolutionized osteoporosis treatment (Liang et al. 2022). As a result, OPG and soluble RANK have been examined as therapeutic targets, and an anti‐human RANKL antibody, denosumab, has been developed. Clinical settings frequently employ denosumab to treat bone abnormalities and cancer‐associated osteoporosis (Udagawa et al. 2021; Zebaze et al. 2016). Postmenopausal women with osteoporosis have also found success with estrogen replacement therapy (Liang et al. 2022). However, these treatments may have limitations or potential side effects. Mandibular osteonecrosis and atypical femoral fractures have been reported as possible complications associated with denosumab use (Anagnostis et al. 2017). Given the possible side effects associated with standard treatments, incorporating complementary and alternative strategies may help mitigate these adverse effects.

Several studies have mentioned that heightened intake of fruits and vegetables positively affects the regulation of bone mineral status (Macdonald et al. 2004; Prynne et al. 2006; Sahni et al. 2009; Tucker et al. 2001). Research has indicated that reactive oxygen species (ROS) may have a major impact on bone resorption (Luo et al. 2025), and certain antioxidants in fruits and vegetables, such as carotenoids, can reduce this oxidative stress (Kiokias and Gordon 2003; Rao et al. 2007; Sahni et al. 2009). Antioxidants are known for deactivating ROS and providing a defense against oxidative damage (Basu et al. 2001; Maggio et al. 2002; Rahim et al. 2015). Inflammatory cytokines can increase the production and maturation of osteoclasts, ultimately leading to the enhancement of bone resorption (Zhou et al. 2019). ROS have been found to influence the expression of OPG and RANKL in osteoblasts. Additionally, ROS inhibit osteoblast differentiation and can initiate apoptosis in osteoblasts (Marcucci et al. 2023; Zhu et al. 2022).

According to an in vitro mechanistic study using mouse bone marrow‐derived macrophages (osteoclast precursors), RANKL may induce osteoclast precursors to generate ROS via pathways involving Ras‐related C3 botulinum toxin substrate 1 (Rac1), nicotinamide adenine dinucleotide phosphate oxidase 1 (Nox1), and tumor necrosis factor receptor‐associated factor 6 (TRAF6) (Lee et al. 2005). Suppressing bone resorption by osteoclasts through inhibition of ROS formation can be carried out by numerous antioxidants, including lycopene.

Lycopene, a carotenoid soluble in lipid and an acyclic isomer of beta‐carotene, lacks provitamin A activity and is primarily found in tomatoes (Marcucci et al. 2023). The potent antioxidant capacity and its mechanism for singlet oxygen scavenging are linked to the beneficial protective role of lycopene (Balali et al. 2025). Previous studies have indicated that lycopene may promote bone mineral density, supporting an interaction between bone integrity and the intake of vegetables and fruits (Alhashemi and Alkhashab 2022; Wattanapenpaiboon et al. 2003). In the past few years, there has been a rise in interest in lycopene as a significant phytochemical that supports human health (Rao et al. 2006). In light of the recent evidence, a reverse correlation between lycopene and carotenoid levels and markers of bone turnover was observed (Kan et al. 2021).

Given the limited evidence synthesizing how lycopene affects bone health through the RANKL pathway, and considering the pathway's critical role in regulating osteoblast and osteoclast activity, we have reviewed the recent literature in this field.

## 2 Description of RANK/RANKL/OPG Pathway Signaling

Placed on the membrane of osteoblasts and activated T cells, RANKL is a homotrimeric protein that may be secreted by diverse cell types, including activated T cells (Yin et al. 2025). Proteolytic cleavage or alternative splicing are mechanisms that result in the release of the protein from the membrane (Ikeda et al. 2001). RANKL proteolytic cleavage is facilitated by matrix metalloproteinases (MMP3 or MMP7) (Lynch et al. 2005) or a disintegrin and metalloprotease domain (ADAM) (Hikita et al. 2006). The majority of stimuli that induce osteoclast development and function stimulate RANKL transcription in osteoblastic stromal cells. RANKL is expressed in several body parts, including mammary glands, thymus, lungs, and lymph nodes; however, this mechanism is diminished in other tissues such as the bone marrow and spleen (Kearns 2008). Additionally, RANKL‐induced osteoclast activation appears to alter HSC migration as part of host defense mechanisms and homeostasis, linking bone remodeling to the control of blood cell formation (Zhang et al. 2023). RANK is a membrane‐integrated protein originally detected in dendritic cells, mature osteoclasts, and osteoclast precursors and belongs to the TNF receptor superfamily (Wu et al. 2020). The synthesis of RANKL by T cells triggers the expression of interferon beta (IFN‐β) in activated osteoclasts via signaling by the Fos proto‐oncogene AP‐1 transcription factor subunit (c‐FOS), which negatively modulates their development (Takayanagi et al. 2002). Interferon gamma (IFN‐γ) produced by T cells and natural killer (NK) cells has been reported to suppress RANK signaling partly through degradation of TRAF6, a crucial adaptor protein that binds RANK to facilitate its signaling (Takayanagi et al. 2000). Furthermore, osteoclast production is complex and appears to depend on certain conditions, such as the bone tissue environment and the abundance of various cytokines that might influence the differentiation of osteoclast precursor cells (OCPs). OCP differentiation is stimulated via nuclear factor of activated T cells 1 (NFATC1) and can be restricted by negative signaling via the OCP‐expressed ligand ephrin B2. Reverse signaling thru ephrinB2 on osteoclast precursors inhibits OCP differentiation by blocking c‐FOS‐dependent activation of NFATC1 after interaction with its receptor EphB4 on osteoblasts. Moreover, EphB4 forward signaling in osteoblasts promotes osteoblast differentiation and bone formation, which further demonstrates the sophisticated regulatory roles of osteoclast–osteoblast interactions other than bone resorption (Zhao et al. 2006).

Besides osteoblasts, several cell types, as observed in the liver, kidney, spleen, and heart, can secrete OPG. Research indicates that B cells may constitute 64% of the total bone marrow OPG (Li et al. 2007). Despite inconsistent research, the majority of investigations point to alterations in RANKL and OPG and, in particular, to a shift in the OPG/RANKL ratio in favor of osteoclastogenesis in conditions with increased bone resorption (Chi et al. 2023).

## 3 Lycopene and Bone Remodeling

The reduced use of interventions for osteoporosis and anti‐osteoporotic medications may be attributed to concerns about their safety. As a result, in Europe, nearly 60% of women vulnerable to osteoporosis are not undergoing proper treatment or any treatment at all, and the use of these treatments has decreased (Kanis et al. 2014). There is a growing interest in therapeutic nutritional approaches with favorable safety profiles. Studies suggest that nutraceutical compounds may be associated with lower bone resorption (Branca 2003). Carotenoids may contribute to bone metabolism regulation and are mainly acknowledged for their antioxidant properties (Yee et al. 2025). The sequence of double bonds dictates their biological functions, including their ability to neutralize free radicals (Milani et al. 2017). Elevated oxidative stress upregulates bone resorption by increasing RANKL expression and signaling, thereby promoting osteoclast differentiation (Lee et al. 2005). Oxidative stress, a key pathogenic indicator in osteoporosis, induces osteocyte apoptosis and alters the expression of particular bone remodeling mediators, including RANKL, sclerostin, and, based on the latest findings, fibroblast growth factor 23, resulting in elevated bone resorption and compromised bone remodeling. Moreover, recent studies implicate dietary antioxidants as potential modulators that mitigate the negative impact of oxidative stress on bone remodeling and osteocytes through the involvement of the sirtuin type 1 enzyme. Multiple findings support the inclusion of antioxidants in innovative therapeutic strategies to optimize the methods used to manage and prevent osteoporosis and oxidative stress‐associated bone diseases. Particularly, anthocyanins, as well as lycopene, oleuropein, resveratrol, various thiol antioxidants, and vitamins, might exhibit protective potential against osteoporosis (Costa‐Rodrigues et al. 2018; Lee et al. 2005; Marcucci et al. 2023).

In recent years, lycopene has garnered significant scientific attention (Silva et al. 2025). Lycopene, predominantly derived from tomato products and tomato itself, ranges from 0.7 to 20 mg per 100 g of fresh tomatoes (Odes‐Barth et al. 2020). Costa‐Rodrigues and colleagues examined how lycopene affects the differentiation and functioning of human osteoclasts and osteoblasts using an in vitro cell culture model involving human peripheral blood mononuclear cells as osteoclast precursors and SaOS‐2 human osteoblast‐like cells. Their findings indicated that lycopene decreases osteoclast differentiation and activation while increasing osteoblast differentiation and proliferation (Costa‐Rodrigues et al. 2018).

Furthermore, a significant decline in indicators of oxidative stress and in markers of bone resorption, particularly the bone resorption marker N‐telopeptide of type I collagen, was observed after 4 months of lycopene intervention in postmenopausal women. A notable rise in serum lycopene levels accompanied this decrease (Mackinnon, Rao, et al. 2011). In 2020, a 12‐week pilot controlled clinical study on postmenopausal women, together with an in vitro examination on osteoblast‐like Saos 2, evaluated the impact of lycopene on osteoblast cells, BMD, and bone turnover markers. The study indicated that lycopene affects several key pathways and bone‐specific markers, such as Wnt/β‐catenin, extracellular signal‐regulated kinase (ERK), type I collagen (COL1A1), runt‐related transcription factor 2 (RUNX2), RANKL, and alkaline phosphatase in SaOS‐2 cells. Lycopene treatment in SaOS‐2‐like cells reduced RANKL, indicating a possible increase in osteoblast differentiation and anti‐resorptive effects. In the clinical arm, the intervention group showed a greater decrease in alkaline phosphatase. Furthermore, the intervention group did not experience as much bone density loss as seen in the control group, implying that lycopene may help prevent short‐term bone resorption in postmenopausal women (Russo et al. 2020). Later in 2020, another study provided an overview of the potential role of lycopene in the prevention of postmenopausal bone loss, focusing on molecular pathways, indicating that lycopene appears to downregulate RANKL expression (Walallawita et al. 2020).

However, studies on lycopene's effects on bone cell metabolism have been short‐term and scarce, and human studies have provided limited insights, with small sample sizes and limited study duration, regarding the impact of lycopene on osteoporosis and its related conditions (Russo et al. 2020).

### 3.1 Effects of Lycopene on Bone Density and Fracture Risk

Lycopene has been associated with improved bone health (Silva et al. 2025). The US population uses tomato‐based foods as a primary nutritional source of lycopene. As evidenced, 13–17 mg of lycopene may be obtained from 6 oz of tomato beverage or 1 cup of prepared concentrated tomato broth. Accordingly, consuming more than 4.4 servings per week of lycopene‐rich products was associated with significantly fewer fractures (Sahni et al. 2009).

In a 12‐week experimental animal study on ovariectomized (OVX) rat models of postmenopausal osteoporosis, lycopene supplementation exhibited dose‐dependent improvements in trabecular bone volume, thickness, cortical parameters, and femoral strength, yielding a bone protective effect comparable to alendronate and indicating the potential role of lycopene in inhibiting bone resorption and preserving bone quality in estrogen‐deficient OVX rats (Ardawi et al. 2016).

Multiple human observational studies have indicated that augmented lycopene intake is connected to a more favorable bone profile and its related markers, observed in both genders (Mackinnon, Rao, et al. 2011; Mackinnon, Venket Rao, and Rao 2011; Sahni et al. 2009). Limited‐scale cross‐sectional research with 33 postmenopausal female participants aged 50–60 years old by Rao et al. demonstrated that higher concentrations of serum lycopene were inversely correlated with the parameters of bone resorption, such as reduced N‐telopeptides of type I collagen and diminished protein oxidation (Rao et al. 2007). Another cross‐sectional study involved 68 men and 137 women from an Australian population who consumed vegetables and fruits rich in phytonutrients. Lycopene intake was associated with increased overall bone mass and improved lumbar spine health in men. Additionally, lycopene, lutein, and zeaxanthin intake were beneficial for premenopausal women. There was also a positive correlation between lumbar spine bone mass and β‐carotene intake in postmenopausal women (Alhashemi and Alkhashab 2022; Wattanapenpaiboon et al. 2003).

An extensive prospective cohort survey with a population of more than 20,000 middle‐aged and elderly adults of both genders demonstrated that higher intake of a carotenoid‐rich diet, particularly of lycopene and combined carotenoids, and their plasma levels were inversely associated with the risk of osteoporotic fractures (Hayhoe et al. 2017). Additionally, according to the 17‐year cohort of the Framingham study, including older men and women, higher lycopene intake was associated with a lower hip fracture risk, most notably in women (Sahni et al. 2009).

### 3.2 Optimizing Osteoblast Differentiation and Function by Lycopene

Bone tissue surfaces are coated with a single‐cell layer of osteoblasts, which form the innermost cell layer in the endosteum and periosteum of cortical bone and envelop all trabecular bone. One of the important steps in the remodeling cycle is the osteoblastic degradation of the unmineralized osteoid found between the osteoblastic cell layer and the mineralized bone. The osteoclast cannot attach to unmineralized bone and can only resorb mineralized parts. Osteoblasts also upregulate the expression of M‐CSF and RANKL (Lerner 2006).

Reducing the differentiation of osteoclasts may be associated with lycopene without affecting their survival. Besides, it facilitates osteoblast development by influencing mitogen‐activated protein kinase (MEK) signaling in both cell types. This occurs through the protein kinase C (PKC) and the nuclear factor‐kappa B (NF‐κB) signaling pathways in osteoblasts (Costa‐Rodrigues et al. 2018; Marcucci et al. 2023). These signaling pathways are central to the regulation of RANKL and OPG expression in osteoblasts. By acting on MEK, PKC, and NF‐κB, lycopene may indirectly reduce osteoclast differentiation through down‐regulation of RANKL and a shift in the OPG/RANKL ratio (Russo et al. 2020).

In 2023, an in vitro study investigated the effects of lycopene on human osteoblasts. The study used the human osteoblast cell line (CRL‐11372) and evaluated the proliferative effects of lycopene using real‐time cell analysis (RTCA) system. In addition, the gene expressions of COL1A1, osteocalcin (OCN), and growth differentiation factor‐5 (GDF‐5) were assessed by quantitative real‐time polymerase chain reaction (qRT‐PCR). Lycopene exhibited dose‐dependent proliferative effects, with specific concentrations enhancing osteoblast proliferation (Bengi et al. 2023).

In an experimental study combining in vivo and in vitro assays in OVX model of osteoporosis rats, daily intake of lycopene enhanced femur bone turnover balance and the functionality of osteoblasts. In vitro, bone marrow‐derived osteoblastic cells from OVX rats exposed to lycopene showed increased alkaline phosphatase activity and higher expression of osteogenic markers, implying an increased osteoblast function (Oliveira et al. 2019).

### 3.3 Mechanisms Linking Lycopene, Oxidative Stress, and RANK/RANKL/OPG Pathway

Oxidative stress plays a crucial role in bone metabolism. ROS production in activated osteoclasts is believed to act as a mediator in bone resorption (Luo et al. 2025). Research highlights a significant positive correlation between excessive bone loss and ROS, and also the progression of many pathological conditions (Costa‐Rodrigues et al. 2018).

Oxidative stress may act as an activator of bone resorption via stimulation of the NF‐κB signaling pathway and various intracellular processes (Luo et al. 2025). A deficiency in NF‐κB can result in reduced osteoclast maturation, leading to reduced bone resorption. Interestingly, research indicates that carotenoid derivatives can lessen the activity of a key regulator of cytokine expression called NF‐κB. This suggests that altering NF‐κB could be one of the pathways through which lycopene provides its protective benefits against osteoporosis (Costa‐Rodrigues et al. 2018; Linnewiel‐Hermoni et al. 2014; Takayanagi et al. 2000). Lycopene's potent antioxidant properties may partially explain its proposed protective effect on bone health, possibly accounting for some of the current findings. Another significant matter pertains to the MEK pathway, as it has been established earlier that oxidative stress affects this pathway (Bai et al. 2004); in osteoclast and osteoblast cell cultures, MEK modulation was also driven by lycopene.

These findings suggest that lycopene may exert its bone‐protective effects through several interconnected pathways. In addition to its effects on NF‐κB and MEK signaling, it also influences antioxidant defenses, intercellular communication, and cell cycle regulation. With respect to the integrated nature of bone remodeling, these combined effects may support proper maintenance of bone homeostasis (Costa‐Rodrigues et al. 2018; Odes‐Barth et al. 2020). Considering that the normal metabolism of osteoclasts and osteoblasts depends on intercellular communications through gap junctions, and thus for bone remodeling, and that lycopene may influence these communications, lycopene could be considered a possible modulator (Costa‐Rodrigues et al. 2018).

In a 2014 experimental animal study, growing female rats (1.5 months old) were given diets containing 0, 50, or 100 ppm lycopene. The findings indicated that lycopene consumption led to elevated bone mass in the body, lumbar spine, and proximal tibial metaphysis in the 100‐ppm group, as well as higher bone alkaline phosphatase (BAP) activity in the 100‐ppm group compared with the 0‐ppm group. Oxidative stress showed no systematic difference between groups; however, higher oxidative stress was correlated with reduced tibial BMD. The outcomes of this study showed that lycopene may accelerate bone formation and reduce the bone resorption process in growing female rats (Iimura et al. 2014).

In a 4‐month randomized controlled trial in postmenopausal women, lycopene supplementation (≥ 30 mg/day from capsules and/or lycopene‐rich juice) reduced oxidative stress and significantly decreased the bone resorption marker N‐telopeptide (NTx) levels. Additionally, restricting lycopene intake in postmenopausal women for 1 month resulted in lower levels of antioxidant enzymes and higher levels of lipid and protein oxidation (Mackinnon, Venket Rao, and Rao 2011).

In addition to typical osteoporosis, diabetic osteoporosis (DOP), recognized as a complication of diabetes, is a widespread disorder involving abnormal bone structure and function (Paschou and Vryonidou 2019). Inflammation is a key pathological process leading to DOP and is also a significant feature in the emergence of insulin resistance and diabetes (Guo et al. 2019). Increased bone resorption and osteoclast differentiation, and suppressed osteoblast maturation can be triggered by inflammatory cytokines, which accelerate bone loss (Huang et al. 2020). Accordingly, one notable target for treating DOP is the OPG/RANKL pathway (Qi et al. 2021).

In an experimental study by Shan Shan Qi et al., streptozotocin‐induced DOP rats exhibited elevated bone formation and resorption markers, with a decrease in the OPG/RANKL level and increased bone resorption. A 10‐week lycopene treatment reduced oxidative stress and inflammatory markers, lowered the heightened bone turnover markers of procollagen type I N‐terminal propeptide (PINP), alkaline phosphatase (ALP), osteocalcin, tartrate‐resistant acid phosphatase 5b (TRACP5b), and C‐terminal telopeptide of type I collagen (CTX‐1) levels, increased the OPG/RANKL ratio, RUNX2 levels, and improved bone strength and trabecular microarchitecture, attenuating diabetes‐related bone resorption in DOP rats (Qi et al. 2021).

**FIGURE 1:**
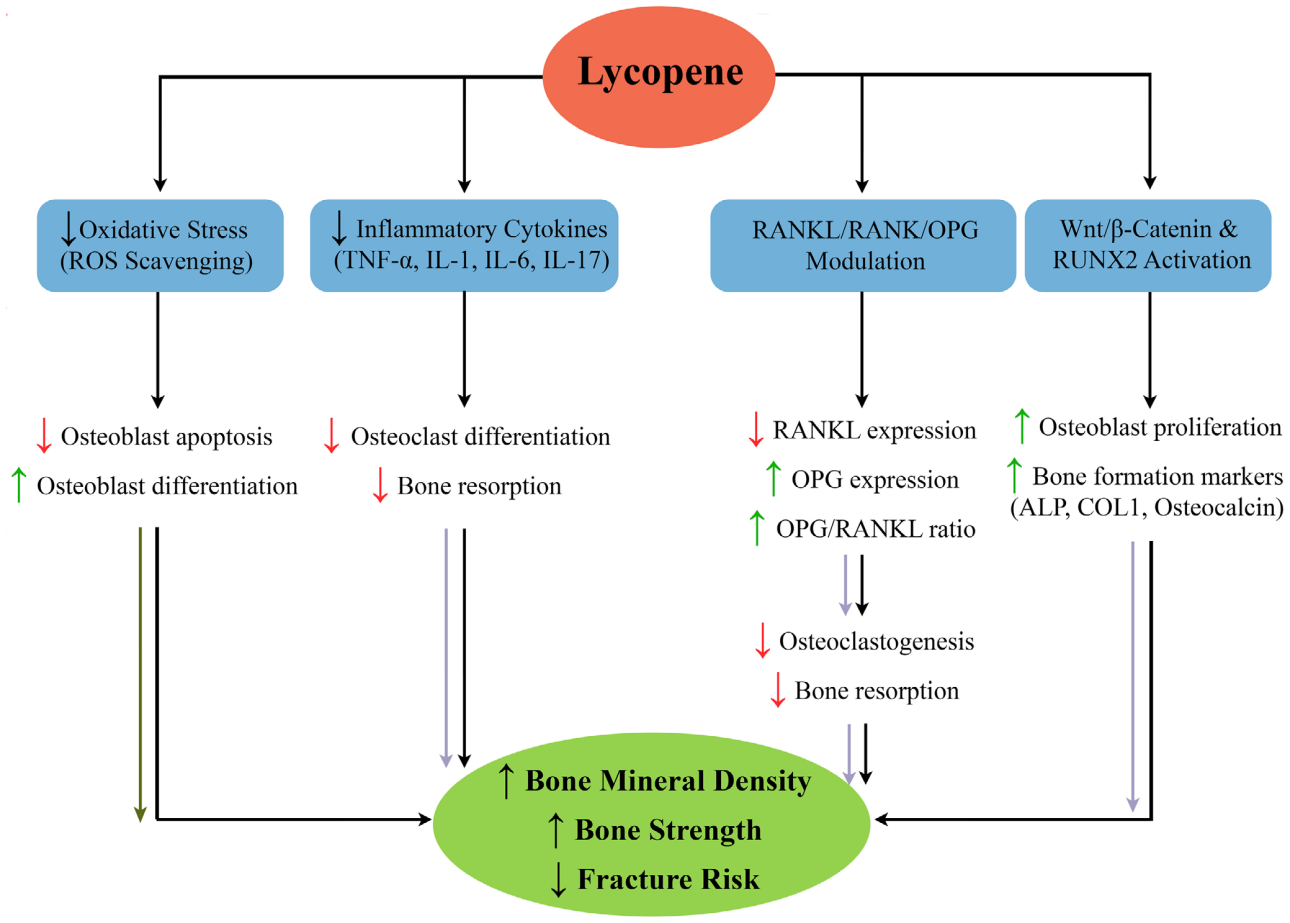
Lycopene‐mediated modulation of bone health via multiple mechanisms. A schematic representation of the mechanisms by which lycopene may improve bone health is shown in the figure. Lycopene is suggested to diminish oxidative stress and inflammatory cytokines, modulate the RANKL/RANK/OPG pathway, and activate Wnt/β‐catenin and RUNX2 signaling, which is proposed to reduce osteoclastogenesis and decrease bone resorption. Dark green arrow indicate pathway supported by human studies, while gray arrows primarily correspond to evidence from rodent experimental models. ALP: alkaline phosphatase; COL1: type I collagen; IL‐1: interleukin‐1; IL‐17: interleukin‐17; IL‐6: interleukin‐6; OPG: osteoprotegerin; RANK: receptor activator of nuclear factor‐kappa B; RANKL: receptor activator of nuclear factor‐kappa B ligand; ROS: reactive oxygen species; RUNX2: runt‐related transcription factor 2; TNF‐α: tumor necrosis factor‐alpha; Wnt: wingless/integrated.

In summary, lycopene has demonstrated potential in enhancing bone health and mitigating osteoporosis through the preceding mechanisms. Notably, the RANKL/RANK/OPG signaling pathway has been frequently highlighted for its significant role in bone health (Figure 1).

## 4 The Role of Lycopene in the RANKL/RANK/OPG Pathway

**FIGURE 2:**
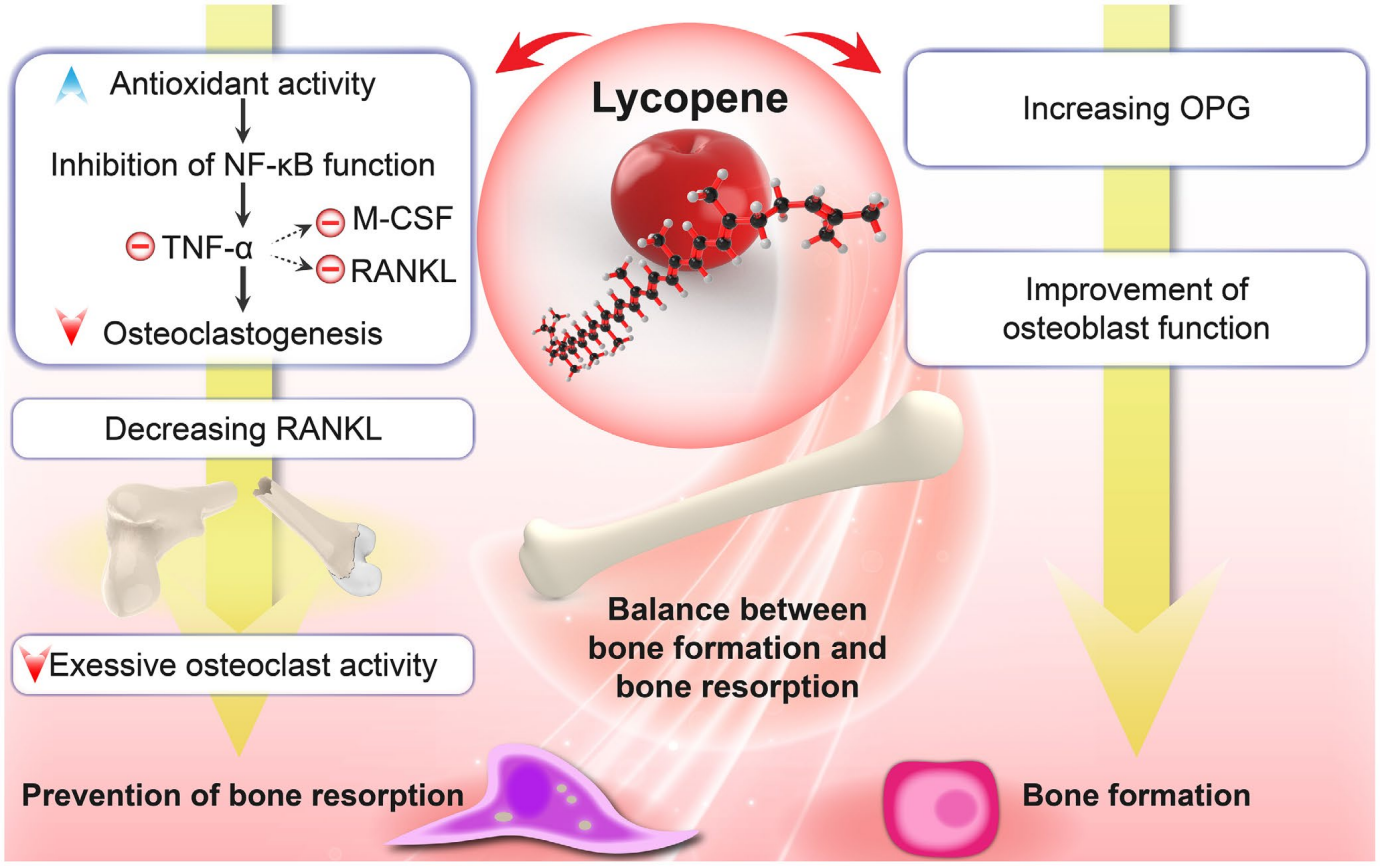
Lycopene‐mediated modulation of the RANK/RANKL pathway in bone remodeling. This figure illustrates the proposed mechanisms by which lycopene may regulate bone health. Lycopene, as a potent antioxidant, may help regulate the balance between bone formation (by osteoblasts) and bone resorption (by osteoclasts) through modulation of oxidative stress‐related pathways, inflammatory signaling, and the RANK/RANKL/OPG pathway. However, these pathways are mainly supported by in vitro and animal studies, and evidence from human research is limited to indirect outcomes, including bone turnover markers and BMD. BMD: bone mineral density; M‐CSF: macrophage colony‐stimulating factor; NF‐κB: nuclear factor kappa‐light‐chain‐enhancer of activated B cells; OPG: osteoprotegerin; RANKL: receptor activator of nuclear factor‐kappa b ligand; TNF‐α: tumor necrosis factor‐alpha.

The metabolic phase is an influential factor in the expression of bone remodeling markers (Shetty et al. 2016). A reduction in antioxidant activity can enhance NF‐κB activation and increase the production of pro‐inflammatory cytokines, particularly TNF‐α, thereby promoting bone deterioration (Hadad and Levy 2012; Kim et al. 2006). TNF‐α enhances osteoclastogenesis by acting synergistically with RANKL, upregulating RANK expression and improving NF‐κB–mediated signaling in osteoclast precursors (Luo et al. 2018). The formation of osteoclasts strongly depends on RANKL and M‐CSF. These two factors are generated by bone marrow stromal cells and osteoblasts throughout bone remodeling (Iñiguez‐Ariza and Clarke 2015). Declining estrogen levels following menopause lead to the downregulation of OPG and the upregulation of RANKL activity, resulting in osteoclastogenesis (Sipos et al. 2009) (Figure 2).

Studies show that prolonged estrogen deficiency induces higher biomarker levels of bone remodeling in postmenopausal women, implying an overall upregulation in bone turnover and greater net osteolysis (Cheng et al. 2022). Diminished estrogen levels result in calcium excretion through the kidneys and reduced absorption in the intestines (Sipos et al. 2009). Numerous bone turnover markers, such as osteocalcin, OPG, and PTH secretion, and the RANK/RANKL system can be triggered by this decrease in calcium levels (Walallawita et al. 2020).

Osteoblasts release osteocalcin, which directly binds to calcium and promotes bone mineralization (Zoch et al. 2016). In women diagnosed with osteoporosis, inadequate calcium and phosphorus stores diminish hydroxyapatite crystal formation, resulting in increased free osteocalcin circulating in the blood (Lewis et al. 2019; Yu et al. 2025).

In multiple cell types, such as bone osteoblasts, activation of the electrophile/antioxidant response element (ARE/Nrf2) transcription system and inhibition of the NF‐κB transcription system are attributed to the oxidative carotenoid derivatives, rather than the preserved carotenoid (Odes‐Barth et al. 2020). Similar yet contrary manifestations on these two transcription systems were identified with synthetic dialdehyde carotenoid derivatives (diapocarotene‐dials), which can be produced through spontaneous oxidation or via chemical or enzymatic oxidation of different carotenoids. There is limited evidence showcasing Diapocarotene‐dials in humans or animals. However, mono‐apocarotenals exhibit comparable yet diminished activity in raw tomatoes (Caris‐Veyrat et al. 2003; Linnewiel et al. 2009; Nara et al. 2001). Moreover, the potential of specific diapocarotene‐dials to trigger the ARE/Nrf2 transcription system and impede the NF‐κB transcription system relies on the chemical responsiveness of their conjugated double bonds. The concentration of electrons around the reactive carbon atoms determines this reactivity (Linnewiel‐Hermoni et al. 2014). It is theorized that oxidized forms of lycopene and other carotenoids may contribute to diminished osteoclast differentiation (Odes‐Barth et al. 2020). Research emphasizes the importance of vegetables and fruits in elevating overall bone health according to their carotenoid content (Zheng et al. 2025). The prominent carotenoid types in plasma include lutein, β‐carotene, and lycopene. Humans tend to take in carotenoids in a non‐selective manner, causing plasma and tissue concentrations to reflect dietary habits of individuals (El‐Sohemy et al. 2002).

NF‐κB activation is among the initial molecular mechanisms following RANK stimulation (Lee et al. 2005; Takayanagi et al. 2000). One of the well‐established characteristics of inflammation is the activation of NF‐κB transcription factors. In this regard, the anti‐inflammatory aspects of lycopene were examined. The study by Joo et al. (2009) revealed that tomato lycopene extract (TLE) has context‐dependent effects on intestinal inflammation: in vitro, TLE reduced LPS‐induced NF‐κB activation in murine splenocytes and IEC‐18 intestinal epithelial cells, leading to lower expression of NF‐κB–dependent pro‐inflammatory mediators such as TNF‐α, IL‐6, and MCP‐1, whereas in vivo, using NF‐κB‐EGFP reporter mice with dextran sulfate sodium (DSS)‐induced acute colitis, dietary TLE worsened disease activity, as reflected by greater weight loss, more severe colonoscopic and histological damage, increased colonic expression of pro‐inflammatory cytokines and chemokines, and enhanced epithelial apoptosis. In another experimental in vitro and in vivo study using lipopolysaccharide (LPS)‐stimulated mouse peritoneal macrophages and a mouse model of inflammation, lycopene exhibited a parallel anti‐inflammatory effect by reducing TNF‐α levels and downregulating NF‐κB activation (Hadad and Levy 2012).

An upregulation of RUNX2 was found by Oliveira et al. (2019) in ovariectomized rats treated with lycopene supplementation. According to previous findings, lycopene at different administered doses augmented RUNX2 mRNA levels, reinforcing the role of lycopene in osteoblast differentiation. The differentiation of mesenchymal stem cells into osteoblasts strongly depends on RUNX2. Notably, RUNX2 regulation induces cell differentiation in the early phases, yet restricts it in the later stages (Mannino et al. 2022; Qi et al. 2021; Russo et al. 2020). Therefore, lycopene may enhance the metabolic activity of osteoblasts by favorably affecting osteoblast differentiation and collagen production (Russo et al. 2020; Sobacchi et al. 2013). A summary of relevant investigations is provided in Table 1.

**TABLE 1:** Summary of evidence on lycopene's effects on bone remodeling.

Study/year	Lycopene source	Model (human/animal)	Lycopene dosage (concentration)	Outcome (effect on RANK/RANKL‐OPG)	Intervention period	References
Wang et al. (2023)	Lycopene extract	Three‐month‐old male SAMP6 mice	50 mg/kg/day	Decreased gene expression levels of RANKL, TRAP, and CTSK	8 weeks	Wang et al. (2023)
Mannino et al. (2022)	N/A	5‐month‐old female SD rats	Lycopene 10 mg/kg. Genistein 5 mg/kg	Increased level of β‐catenin, Wnt5a, and Nrf‐2 expression, stimulating osteoblast function, enhanced level of bALP and RUNX2	120 days	Mannino et al. (2022)
Qi et al. (2021)	Lycopene powder extracted from tomato, purity ≥ 98	8‐week‐old SPF grade male SD rats	50 mg/kg/day. 100 mg/kg/day. 150 mg/kg/day	Upregulated levels of the OPG/RANKL ratio. Upregulated levels of RUNX2, bone, and serum OPG expression. Down‐regulated expression of RANKL	10 weeks	Qi et al. (2021)
Russo et al. (2020)	Tomato sauce	Healthy postmenopausal women	150 mL/day of a tomato sauce	Significant decrease in BAP	12 weeks	Russo et al. (2020)
Odes‐Barth et al. (2020)	Purified crystalline lycopene (> 97%) of tomato extract	RAW264.7 murine monocyte macrophage‐like cells	10 μM	Inhibition of RANKL‐induced osteoclast differentiation by partially oxidized lycopene	2–3 days	Odes‐Barth et al. (2020)
Russo et al. (2020)	Tomato sauce	Human SaOS‐2 cells	5 and 10 μM	Increased mRNA expression levels of COL1A and RUNX2, and decreased RANKL mRNA expression levels	24 h	Russo et al. (2020)
Oliveira et al. (2019)	Lycopene powder (concentration of 1 μmol/L)	OVX Wistar rats	10 mg/kg/day	Reduced bone resorption. Significant increase in cell proliferation	8 weeks	Oliveira et al. (2019)
Matsumoto et al. (2018)	βcarotene containing diet (including lycopene)	Seven‐week‐old female ddY mice	0.025%. 0.05%. 0.25%	Increased mRNA expression of OPG	3 weeks	Matsumoto et al. (2018)
Ardawi et al. (2016)	N/A	6‐months‐old female Wistar rats with OVX and SHAM surgery	15 mg/kg/day. 30 mg/kg/day. 45 mg/kg/day	Elevated BMD and BMC. Decreased bone resorption when measured against the control group. Decreased bone resorption	12 weeks	Ardawi et al. (2016)
Mackinnon, Rao, et al. (2011)	Supplements and food resources containing lycopene (based on a given list)	Healthy postmenopausal women aged 50–60 years old	Limitations of lycopene to 3.5 mg/day initially and 0.13 mg/day after 1 month	Heightened levels of NTx. Significant reduction of serum lycopene and GPx	4 weeks	Mackinnon, Rao, et al. (2011)

Abbreviations: bALP: bone alkaline phosphatase; BAP: bone alkaline phosphatase; BMC: bone mineral content; BMD: bone mineral density; COL1A: collagen type I alpha 1 chain; CTSK: cathepsin K; ddY: Doyen dwarf Y; GPx: glutathione peroxidase; Human SaOS‐2 cells: human osteoblast‐like cells; Nrf2: nuclear factor erythroid2‐related factor 2; NTx: N‐terminal telopeptide; OPG: osteoprotegerin; OVX: ovariectomized; RANK: receptor activator of nuclear factor‐kappa B; RANKL: receptor activator of nuclear factor‐kappa B ligand; RUNX2: runt‐related transcription factor 2; SAMP6: senescence‐accelerated mouse prone 6; SD rats: Sprague–Dawley rats; SPF: specific pathogen free; TRAP: tartrate‐resistant acid phosphatase; Wnt5a: wingless‐type MMTV integration site family, member 5A.

The bone marrow cell microenvironment is altered in diabetic conditions (Wu et al. 2024). Osteoblasts or adipocytes can be derived from MSCs. Prolonged hyperglycemia enhances adipogenic differentiation and decreases osteogenic differentiation of MSCs, thereby altering the bone marrow microenvironment (Huang et al. 2019).

Studies revealed that diabetic rats exhibited lowered OPG and heightened RANKL expression, leading to a decreased OPG/RANKL ratio, which reflected enhanced bone resorption relative to bone production. Another contributor to the regulation of bone formation, RUNX2, contributes to osteoblast differentiation by controlling the gene expression of osteoblast‐specific extracellular matrix proteins, thereby promoting osteoblast differentiation and bone formation (Ruiz et al. 2018). According to the current studies, an upregulation of RUNX2 expression in diabetic rats was observed following lycopene intake, which can lead to improved BMD and elevated bone formation (Oliveira et al. 2020; Zheng et al. 2018). Evidence from observational human studies has shown that diabetes is associated with elevated levels of bone turnover markers, reduced bone mineral density, and increased fracture risk (Onyenekwu et al. 2017; Sherk et al. 2020). However, there are very limited data from long‐term human studies investigating the diabetes‐related changes in bone turnover markers. Further in‐depth clinical exploration is needed to substantiate the possible therapeutic role of lycopene in metabolic bone diseases.

## 5 Conclusion

Current findings suggest a possible association among the RANKL/RANK/OPG signaling pathway, lycopene, and various indicators of bone health. Notably, the influence of lycopene on bone health has attracted attention owing to its potential role as a dietary factor in modulating osteoclastic activity and bone metabolism via the RANK/RANKL/OPG signaling pathway. Lycopene may contribute to the downregulation of RANKL transcription, thereby hindering the development and activation of osteoclasts, the cells primarily responsible for bone resorption. This inhibition is closely linked to the preservation of bone mineral density and microarchitectural integrity, especially in conditions characterized by elevated osteoclast activity, such as postmenopausal osteoporosis. In addition, the antioxidant properties of lycopene may confer further protective effects on bone health by reducing oxidative stress and affecting the RANK/RANKL pathway, which is increasingly acknowledged as a key modulator in osteoporotic changes and impaired bone formation.

However, most related studies are based on in vitro and animal evidence, which can be mechanistically informative but cannot establish direct causality or optimal dosing in humans. Moreover, human studies are predominantly short‐term investigations or small pilot trials, mostly conducted in vulnerable populations, including postmenopausal women, concentrating on surrogate markers such as bone turnover or oxidative stress indicators rather than long‐term effects like fractures. Many observational findings may also be confounded by overall dietary patterns. Considering the variations in lycopene source, skeletal sites, dose, duration, and different demographic groups, a firm dose–response recommendation is not defined yet. Additionally, lycopene bioavailability is influenced by multiple factors, including food processing and dietary fat intake, which are not sufficiently addressed in the literature. Moreover, although lycopene has been associated with modulation of oxidative stress, NF‐κB signaling, and the RANK/RANKL/OPG pathway, direct causal mechanisms in humans remain uncertain. Consequently, given the multiple biological effects attributed to lycopene, further clinical investigations are required to illuminate how lycopene is connected to bone health through modulation of different interconnected mechanistic pathways.

## Author Contributions

**Sadegh Jafarnejad:** supervision, project administration. **Helia Esmaili:** conceptualization, writing – original draft, visualization. **Negar Mosayeb:** writing – review and editing. **Nazanin Asgari:** writing – review and editing.

## Funding

The authors have nothing to report.

## Disclosure

No generative AI tools were used for preparing the manuscript or figures.

## Conflicts of Interest

The authors declare no conflicts of interest.

## Data Availability

Data sharing is not applicable to this article, as no new data were created or analyzed.
